# Implementing a function-based cognitive strategy intervention within inter-professional stroke rehabilitation teams: Changes in provider knowledge, self-efficacy and practice

**DOI:** 10.1371/journal.pone.0212988

**Published:** 2019-03-11

**Authors:** Sara E. McEwen, Michelle Donald, Katelyn Jutzi, Kay-Ann Allen, Lisa Avery, Deirdre R. Dawson, Mary Egan, Katherine Dittmann, Anne Hunt, Jennifer Hutter, Sylvia Quant, Jorge Rios, Elizabeth Linkewich

**Affiliations:** 1 St. John’s Rehab Research Program, Sunnybrook Research Institute, Toronto, Ontario, Canada; 2 Department of Physical Therapy, University of Toronto, Toronto, Ontario, Canada; 3 Rehabilitation Sciences Institute, University of Toronto, Toronto, Ontario, Canada; 4 North & East GTA Stroke Network, Sunnybrook Health Sciences Centre, Toronto, Ontario, Canada; 5 Avery Information Services Ltd., Orillia, Ontario, Canada; 6 Department & Occupational Science & Occupational Therapy, University of Toronto, Toronto, Ontario, Canada; 7 Rotman Research Institute, Baycrest, Toronto, Ontario, Canada; 8 School of Rehabilitation Sciences, University of Ottawa, Ottawa, Ontario, Canada; 9 Bloorview Research Institute, Holland Bloorview Kids Rehabilitation Hospital, Toronto, Ontario, Canada; 10 Faculty of Medicine, University of Toronto, Toronto, Ontario, Canada; 11 Practice-Based Research, Sunnybrook Research Institute, Toronto, Ontario, Canada; Foundation IRCCS Neurological Institute C. Besta, ITALY

## Abstract

**Background:**

The Cognitive Orientation to daily Occupational Performance (CO-OP) approach is a complex rehabilitation intervention in which clients are taught to use problem-solving cognitive strategies to acquire personally-meaningful functional skills, and health care providers are required to shift control regarding treatment goals and intervention strategies to their clients. A multi-faceted, supported, knowledge translation (KT) initiative was targeted at the implementation of CO-OP in inpatient stroke rehabilitation teams at five freestanding rehabilitation hospitals. The study objective was to estimate changes in rehabilitation clinicians’ knowledge, self-efficacy, and practice related to implementing CO-OP.

**Methods:**

A single arm pre-post and 6-month follow up study was conducted. CO-OP KT consisted of a 2-day workshop, 4 months of implementation support, a consolidation session, and infrastructure support. In addition, a sustainability plan was implemented. Consistent with CO-OP principles, teams were given control over specific implementation goals and strategies. Multiple choice questions (MCQ) were used to assess knowledge. A self-efficacy questionnaire with 3 subscales (Promoting Cognitive Strategy Use, PCSU; Client-Focused Therapy, CFT; Top-Down Assessment and Treatment, TDAT) was developed for the study. Medical record audits were used to investigate practice change. Data analysis for knowledge and self-efficacy utilized mixed effects models. Medical record audits were analyzed with frequency counts and chi-squares.

**Results:**

Sixty-five health care providers consisting mainly of occupational and physical therapists entered the study. Mixed effects models revealed intervention effects for MCQs, CFT, and PCSU at post intervention and follow-up, but no effect on TDAT. No charts showed any evidence of CO-OP use at baseline, compared to 8/40 (20%) post intervention. Post intervention there was a trend towards reduction in impairment goals and significantly more component goals were set (z = 2.7, p = .007).

## Introduction

While it is well established that implementing even a relatively simple practice change can be challenging, few studies have examined the implementation of a complex intervention in inter-professional, multi-site environments. A group of knowledge users and researchers in a large urban centre in Canada embarked on such a project in stroke rehabilitation, to address two concerns: One, persons with cognitive impairments following a stroke had decreased access to inpatient rehabilitation [1], despite evidence of its benefits for them [2]; and two, when rehabilitation was accessed, persons with cognitive impairment received services based on outdated impairment-reduction models, rather than recommended function-based approaches [3]. There was evidence to suggest that these two issues could be traced back to a reported lack of skills and knowledge on the part of stroke rehabilitation teams to work with persons with cognitive impairments [3]. A multi-faceted, supported, knowledge translation (KT) initiative, targeted specifically at the inter-professional application of the Cognitive Orientation to daily Occupational Performance (CO-OP) approach in inpatient stroke rehabilitation teams at five freestanding rehabilitation hospitals [4] was implemented and evaluated. CO-OP is a person-centred treatment approach, framed around the use of cognitive strategies, which is aligned with Canadian Stroke Best Practice Recommendations for cognitive rehabilitation [5]. The project, called CO-OP KT, has three embedded studies related to client outcomes; provider knowledge, self-efficacy, and practice; and health system level changes in access to inpatient rehabilitation for persons with cognitive impairments. The protocol for the larger project has been published [6]. The focus of this paper is on findings related to changes in providers’ knowledge, self-efficacy, and practice to implement a cognitive-strategy-based approach following the CO-OP KT intervention.

### The clinical intervention: CO-OP

CO-OP is a functional, client-goal-focused, problem solving approach that is associated with improved function, activity performance, participation, and self-efficacy among persons with a wide range of conditions, including cognitive impairment following a stroke [7–13]. In the stroke population, several studies have demonstrated these benefits, including randomized controlled trials in inpatient rehabilitation and sub-acute rehabilitation. Anecdotally we are aware that CO-OP is used by several health professional disciplines, including occupational therapy, physical therapy, and therapeutic recreation, and one publication provides preliminary evidence that the inter-professional application of CO-OP is feasible [14].

CO-OP is described as a top-down approach that prioritizes whole-task practice of functional activities (e.g. practicing walking, dressing, or remembering a grocery list). This is in comparison to bottom-up approaches that prioritize impairment reduction, such as strengthening or memory exercises done with the premise that functional activity will develop once the impairments are remediated. CO-OP has seven key features, five of which are considered essential elements: client-chosen functional goals, dynamic performance analysis, cognitive strategy use, guided discovery, and use of enabling principles [15]. In the first session, the client and a rehabilitation clinician work together, using the Canadian Occupational Performance Measure (COPM) [16], to select personally-important activities with which the client is having performance issues. These activities form the basis of the rehabilitation goals and become the focus of the CO-OP intervention. In the next meeting, the client is taught a global cognitive strategy (Goal-Plan-Do-Check [17]) that is then used in all subsequent sessions as the main problem-solving framework to facilitate skill acquisition/goal attainment. The client identifies a Goal, and then is guided by the clinician to make a Plan, then Does the Plan and finally Checks to see if the plan worked. The clinician uses guided discovery rather than explicit instruction to *guide* the client to analyze the task to be performed and to *discover* strategies that are specific to that client’s individual performance challenges with that particular activity. A detailed description of CO-OP’s administration procedures is available in a publication by Polatajko and Mandich, 2004 [4], and current thinking on its theoretical foundations and essential elements are available in a book published in 2017 [18], which also includes details regarding adaptations for persons who have experienced stroke [19].

Basic CO-OP training is provided through a 3-day standardized workshop that is overseen by the International CO-OP Academy (co-opacademy.ca). CO-OP instructors, who are certified and monitored by the Academy provide the standardized workshop. It consists of two full days of instruction and practice in CO-OP, followed by a consolidation day approximately two to six months after participants had a chance to practice using CO-OP in their clinical settings. During the consolidation day, participants present and discuss cases, and form goals and plans for ongoing implementation of CO-OP.

### Desired practice change, implementation considerations, and proposed solutions

The desired change was that stroke rehabilitation team members learn to use the CO-OP Approach, and incorporate it into their individual and team practices. We did not set a target for level of CO-OP use (e.g., use of CO-OP with all clients with stroke or with 50% of clients with stroke), just that it be used as much as feasible, and that, once learned, it would be used by participants for most clients, including those with cognitive impairments.

We anticipated that the uptake of CO-OP within an inter-professional inpatient stroke rehabilitation team would require careful consideration for implementation. The first consideration was that CO-OP was originally designed for use by a single clinician, primarily occupational therapists, usually in an outpatient setting. Although CO-OP has been used successfully in inpatient stroke rehabilitation [7, 11], and by occupational and physical therapists working together [14], it had not been previously used by a full inter-professional team in an inpatient setting. Thus, the exact processes for an inpatient, inter-professional application were not known, and would have to be worked out by the individual participating teams and research team.

The second consideration was that using CO-OP requires health professionals to move away from traditional bottom-up impairment-reduction therapies and towards a top-down, experiential learning-based approach. Stroke rehabilitation has traditionally focused on impairment-level treatments, such as reducing muscle tone, improving balance, or improving attention and memory, with the belief that altering body functions and structures will eventually translate to functional improvements. There is little empirical evidence to suggest this to be true [2, 20], and more holistic experiential learning-based theories have emerged, arguing that functional recovery results from learning-dependent neuroplasticity, which is optimized through a complex interaction of cognitive, psychological, and contextual factors that consider an individual’s specific needs [21]. CO-OP asks that a therapist shift away from a focus on, for example, exercises that improve a client’s memory, and instead focus on helping the client to develop strategies to support function, such as determining the need to use a shopping list to remember items to buy while carrying out their self-identified goal of grocery shopping. While impairment reduction aims still exist, they are moved to the background of treatment, while meaningful, functional goals are moved to the foreground. This shift of focus to task-specific training of activities that are important to the person is in line with best practice recommendations [22], but does require a shift from impairment focus that is deeply ingrained in stroke rehabilitation [23].

The third consideration was that using CO-OP requires that the functional treatment goals come directly from the client. While client-centred goal setting has been the focus of publications and conversations for decades [24], its use in practice is still inconsistent [25]. In addition, therapists frequently cite cognitive impairment as a significant barrier to client goal setting [26, 27].

The fourth consideration was that a main therapeutic technique in CO-OP is guided discovery. Guided discovery requires that health professionals guide clients to discover their own solutions to performance problems by asking questions and coaching, rather than providing them direct instruction or relying on manual facilitation techniques of which the client is unaware. Urquhart and Skidmore [28] reported that over 95% of the instructions given by occupational and physical therapists and speech-language pathologists to persons with stroke are directive rather than guided, indicating the enormity of this desired practice change.

Finally, and perhaps most importantly, three of the considerations, using a top-down learning-based approach, having client goals be the direct focus of therapy, and using guided discovery, all require that the therapist embrace shared decision making and shift considerable control of therapeutic goals and intervention to the client. Numerous barriers to using shared decision making exist for health care providers, including attitudes, lack of familiarity and experience with the process, lack of continuity of care, client-provider relationships, lack of resources, and time [29].

To address these anticipated challenges, we began with an equal knowledge user and researcher partnership, and used the Knowledge to Action (KTA) framework as a foundation for our KT program development [30]. The KTA framework is well-known and has been used successfully in several projects [31–35], including in stroke rehabilitation [36]. The KTA framework takes a macro view of KT, consisting of a central knowledge creation cycle and a concurrent action cycle that outlines several action phases to guide the application of knowledge in practice. It helped us to structure our initial problem solving regarding the specific KT challenge, the local context, and potential barriers. We aimed for an active, multi-component KT approach to effect widespread, sustained practice change [37–39]. We incorporated inter-professional collaboration, which promotes effective working relationships among health care providers from different disciplines and their clients and enables optimal health outcomes by building on the foundational elements of “respect, trust, shared decision making and partnerships” [40]. We addressed health system components (e.g., engagement of decision makers), as they are believed to be important in moving evidence to practice in complex environments, particularly when shifts in culture, attitudes, and behaviour are required [41, 42]. It is also well understood that support from management is a significant implementation facilitator [37, 38, 43], and that it is closely linked to more receptive staff attitudes [38]. Finally, to further address the enormity of the required paradigm shift, CO-OP KT was framed within the CO-OP process to give as much individual and site control over changes as possible. Within this approach, individual sites were asked to set implementation goals that made sense within their context, an implementation facilitator (role described below) guided discovery to help teams develop, implement, and check plans, and teams were guided to modify plans when they were not achieving the desired result.

### Objectives

The overall aim of this study was to estimate changes in rehabilitation clinicians’ knowledge, self-efficacy, and practice related to implementing a complex, client-centred, cognitive-strategy-based treatment approach following the multi-faceted KT initiative known as CO-OP KT. The specific research objectives were as follows:

1. Describe research participants’ knowledge and self-efficacy related to implementing a cognitive-strategy-based treatment approach, including by discipline and research site, at baseline, post-intervention, and at a 6-month follow-up;
2. Estimate the effect of CO-OP KT on post-intervention and 6-month follow-up changes in rehabilitation clinicians’ knowledge and self-efficacy;
3. Estimate the amount of clinical practical use of a cognitive-strategy-based treatment approach before and after the CO-OP KT intervention, using indicators from medical records.

## Methods

This study is one of three sub-studies within a larger project; the current study examines health care provider outcomes, another examines health system level outcomes, and a third examines patient level outcomes. The study involving patient outcomes was registered in 2016 on NIH’s website clinicaltrials.gov, registration #NCT02597569. A single arm pre-post-follow up study was conducted with stroke rehabilitation clinicians from five publicly-funded, freestanding rehabilitation hospitals in a Toronto, Ontario, Canada. Research Ethics Board approval was obtained from the Mount Sinai Hospital Research Ethics Board, the Providence Healthcare Research Ethics Board, the Sunnybrook Health Sciences Centre Research Ethics Board, the University Health Network Research Ethics Board, and the West Park Healthcare Centre Joint Research Ethics Board. All clinicians who were invited to complete the questionnaires provided informed consent. Patient data were extracted from medical charts and the ethics committees waived consent.

Table 1 provides an overview of the relative timing of assessments and intervention elements.

**Table 1:** Key assessment and intervention time points.

	Key time point	Dates	Activity and Description
A1	Baseline chart audit– 6 months before CO-OP KT	April 2016	
T1	Pre CO-OP KT assessment	September 29 to October 24, 2016	**MCQ and SERTA Baseline:** Email links were sent on Sept. 29, 2016 and participants were given until the morning of the workshop (Oct. 24, 2016) to complete the surveys.
	CO-OP workshop Part I (two days of instruction and practice)	October 24–25, 2016	
T2	Post CO-OP workshop assessment	October 26 to November 18, 2016	**MCQ and SERTA Post 1:** Email links were sent on Oct. 26, 2016 and participants were given until Nov. 18, 2016 to complete the surveys.
	CO-OP KT implementation support period and infrastructure support	November 2016 to February 2017	
T3	CO-OP Workshop Part II (Consolidation Sessions) and Post CO-OP KT assessment	March to April 2017	**MCQ and SERTA Post 2:** Each site was sent email links immediately following their Consolidation Session and given until Apr. 13, 2017 to complete the surveys. The Consolidation Sessions were scheduled throughout Mar. 2017, so sites had between 7 and 9 weeks to complete them.
A2	Post CO-OP KT chart audit—within the month following CO-OP KT	April 2017. Chart audits for each site were initiated once their Consolidation Session and Post CO-OP KT assessment was complete	
	CO-OP KT sustainability plan	Initiated April 2017, ongoing	
T4	6 months post CO-OP KT	October 2017	**MCQ and SERTA Post 3:** Email links were sent on Sept. 18, 2017 and participants were given until Oct. 20, 2017 to complete the surveys.

### Recruitment/Participants

Participants were recruited from each of the five inpatient stroke rehabilitation sites. All healthcare professionals providing direct care were invited by email to participate in the CO-OP KT. The research team recommended that each site group include all or most occupational and physical therapists and speech-language pathologists, at least one nurse, and any other team members who showed interest (e.g. occupational and physical therapist assistants, social workers) and who had a direct client care role. As all members were invited, no sample size calculations were required, however, we planned for an average of 15 healthcare professionals per site. Funds were offered for replacement of staff attending the initial 2-day workshop.

As is typical in institutional settings, and because the training necessitated a substantial number of team members be absent from client care for two full days, team managers made the final decisions about who could and could not participate, in consultation with the research team. For example, in some rare cases, the manager did not approve a team member attending CO-OP training if they could not find a replacement professional to provide coverage for his or her clients. In some cases, the research team reviewed the proposed list of attendees and made suggestions to the manager about removing someone from the list (e.g. a clinical educator who did not provide direct client care) or additions to the list (e.g. a nurse to round out the inter-professional team).

### The CO-OP KT intervention

The CO-OP KT intervention consists of the following components:

1. The standardized initial *CO-OP 2-day workshop*, which includes two full days of instruction and practice, led by an experienced and certified CO-OP instructor who was not part of the research team;
2. A 4-month *implementation support* period that included the following:
  - Support of an implementation facilitator to promote attainment of site-specific implementation goals through bi-weekly face-to-face meetings with participants, interim email and phone support, and site-specific tailoring of implementation support processes;
  - Implementation materials, including an implementation workbook, posters, and information cards; and
  - Access to a CO-OP instructor who was able to visit sites once or twice to answer specific questions.
3. The established CO-OP training workshop *consolidation session*, run in this case as a half-day session for each team separately at the end of the support period, led by a certified CO-OP instructor who was part of the research team (SM); and
4. *Infrastructure support*:
  - Linkages with an existing stroke network knowledge translation infrastructure, including a virtual community of practice, regular engagement of team leaders and decision makers; and
  - Access to a short (approximately thirty minutes to complete) introductory online CO-OP module for all team members who did not attend the workshop, called CO-OP I (http://ot.utoronto.ca/clinical-community-alumni/continuing-education/coop/).

In addition to the formal CO-OP KT program (2-day workshop, implementation support, consolidation session, infrastructure support), we also developed a *sustainability plan* that was put into action after the formal CO-OP KT intervention period had ended. The sustainability plan included the following components:

1. Establishment of site champions;
2. Bi-monthly teleconferences with site champions facilitated by the stroke network regional education coordinator;
3. Quarterly teleconferences with the site leaders facilitated by the stroke network regional director; and
4. Free access to an online version of the CO-OP workshop and consolidation session for new team members (http://ot.utoronto.ca/clinical-community-alumni/continuing-education/coop/).

### Outcome indicators

Three constructs were measured in this study: knowledge, self-efficacy, and practice change. The outcome measures and indicators for each are described below, and Table 1 provides an overview of key assessment and intervention time points. Knowledge and self-efficacy were assessed on a slightly different timeline than practice. Knowledge and self-efficacy were assessed at the following time points: pre-intervention (T1); post-workshop (T2); post CO-OP KT intervention (including implementation support and consolidation sessions but excluding the sustainability plan), approximately 4 months after T2 (T3); and 6-month follow-up (T4). Practice was assessed by medical record audits, which were conducted approximately six months before the start of CO-OP KT (A1), and within the month after the consolidation sessions (A2).

Knowledge was assessed using a subset of a bank of multiple-choice questions (MCQs) that had previously been developed for use in the established CO-OP workshops. The 26 MCQs covered the CO-OP definition, objectives, and details about the approach’s seven key features. At each assessment time point a research staff member sent each participant a link to the survey with a unique and unidentifiable code to use when each participant logged in. Other than site and discipline, no personal identifiers were collected.

Self-efficacy was assessed using a 21-item, 0 to100-point survey developed based on Bandura’s guidelines [44], named the Self-Efficacy Rating for Top-down Approaches (SERTA). The SERTA was developed specifically for use in this study. Its 21 items came from CO-OP’s treatment fidelity checklist [45] and were refined with input from the research team and a group of certified CO-OP instructors. Three subcomponents were established using principle component analysis and expert opinion: 1) Top-Down Approaches and Assessments (TDAT), 2) Client-Focused Therapy (CFT), and 3) Promoting Cognitive Strategy Use (PCSU). As with the MCQs, a research staff member sent each participant a link to the survey at each assessment time point with a unique and unidentifiable code to use when the participant logged in. Other than site and discipline, no personal identifiers were collected.

The number of MCQ and SERTA items and the 4- and 6-month time lags between T2 and T3 and T3 and T4 respectively were felt by the research team to be sufficient to mitigate and potential recall bias. Practice was assessed by medical record audits. The main inclusion criterion was charts of clients who had been discharged home from a high intensity stroke rehabilitation program 6 months before or within the month after CO-OP KT. The post-intervention audit included an additional criterion that the client had to have received treatment from at least one provider who participated in CO-OP KT. At each time point, medical records departments were asked to retrieve 12 records within the appropriate discharge period, and the reviewers selected the first eight charts that met the criteria. The records were reviewed for documentation related to treatment goals; whether the goals comprised of a functional activity (e.g. independence with upper body dressing), a component of a functional activity (e.g. reaching), or an impairment-reduction goal (, e.g. increase in arm strength); clear evidence of client involvement in the goal-setting process; and use of the CO-OP Approach or its elements in treatment. Working definitions (Table 2) were developed for each item coded in the medical record audit, and two to three members of the research team made consensus-based decisions about how an item should be coded when the coder was unsure.

**Table 2:** Working definitions for goal types.

Type of Goal	Definition
Functional goals	Goals aimed at increasing the client’s independence with performance of activities of daily living such as “toileting with minimal assistance” or “remembering therapy schedule”. These goals may also involve tasks or activities that are unique to the client’s lifestyle, such as “typing on a keyboard” or “organizing and filing paperwork”.
Impairment goals	Non-functional goals aimed at increasing capacity of or normalizing impaired body structures (increasing strength, decreasing tone) or bodily activities (attention, balance) or raising scores of non-functional scales (e.g. improve Berg Balance Score, improve MoCA score).
Component goals	Goals aimed at increasing ability in one element of an activity, task, or skill but without a specific outcome or endpoint (e.g. grasping) or with a nonfunctional endpoint (e.g. grasping cones, sorting coins).
Client-centred goals	Goals that seem to have been developed by or in collaboration with the client. Evidence that the goal is client-centred may be: The goal or aspects of the goal is written in quotes to indicate it is the client’s own words; The goal is not a typical in-patient rehab activity (e.g. transferring, walking with walker, upper body dressing, etc.) but has elements that seem unique (e.g. walk on a slope, put on winter jacket); There is some annotation in the chart to indicate that the goal is important to the client (e.g. it sounds like a typical walking goal, but there is evidence from progress notes or team meetings that the client is focused on walking independently).

### Data analysis

Data were cleaned and verified. Descriptive statistics were compiled for all outcomes. To examine post-intervention and follow-up changes relative to baseline scores for the SERTA and MCQ and to understand the incremental effects of the workshop and the support period on MCQ and SERTA scores while allowing for variability among participants and sites, mixed effects models were used. All team members with at least two assessments available were included in the analysis. Dummy variables were created to allow the assessment of incremental change at each time point. To examine the effectiveness of the CO-OP KT intervention as a whole (2-day workshop + implementation support + consolidation session + infrastructure support), we initially examined the pre-intervention (T1), post-intervention (T3) and follow-up assessments (T4), while excluding the post-2-day-workshop (T2) evaluations. Within-subject dependence was modeled using participant-level random effects at baseline, intervention and follow-up. Site-level random effects were also modelled at each time point. Random effects < 0.01 were removed from the model, resulting in improved Bayesian information criterion (BIC) statistics, and no changes to the fixed effects in all cases. Model fit was assessed by checking the normality of the model residuals and random effects. To determine the relative contributions of the workshop and the support period to the overall intervention effects, secondary models were fit incorporating the post-workshop data and examining the distinct changes from T1 to T2 and T2 to T3 (whereas the primary analysis examined the combined T1 to T3 change).

Results of the medical record audit were analyzed with frequency counts, and pre and post comparisons were made with chi-square analyses. Statistical analyses were completed using SPSS version 24 and SAS version 9.4.

## Results

**Fig 1:**
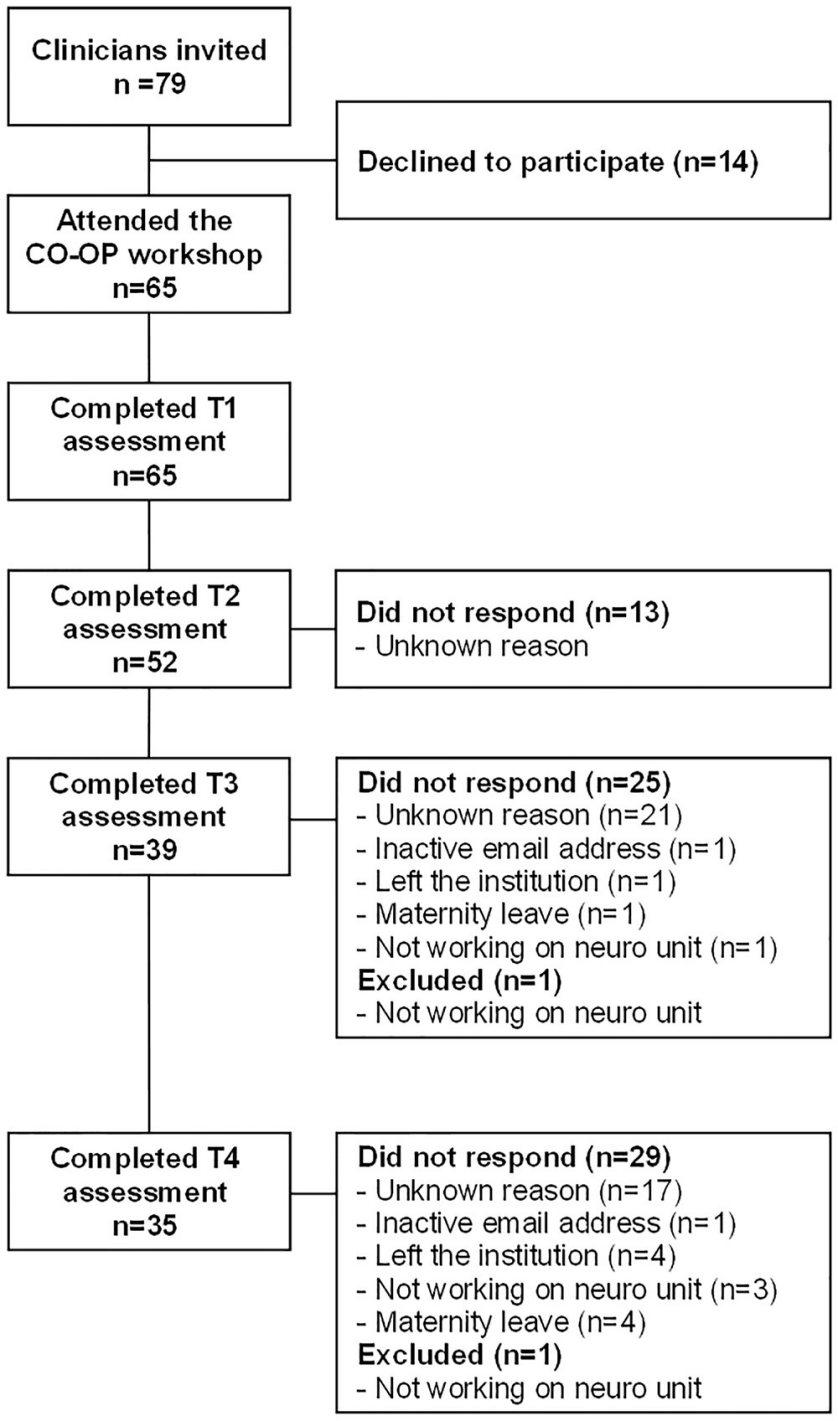
CONSORT diagram.

The flow of health care provider participants in this study are displayed in the CONSORT diagram, Fig 1. Sixty-five health care providers entered the study at T1. Regarding responses to the MCQ and SERTA, 20% (n = 13) withdrawal rate occurred by T2 (within the month following the workshop), with an additional 20% (n = 13) withdrawal at T3 and 6% (n = 4) more at T4. Thus, the overall withdrawal rate by the T4 follow-up was 46%. Of the 46%, 12 participants (40%) were not working at the site (e.g., changed jobs, went on leave, etc.), while 18 participants (60%) were still part of the team but did not respond to the surveys for unknown reasons.

It should be noted that one site had complex organizational restructuring issues during the initial recruitment that resolved before the 2-day workshop, but not in time to allow a full complement of participants to attend the training. A condensed 1-day workshop was therefore held for six additional participants approximately one month later. The additional six participants are included in Fig 1.

Table 3 provides the distribution of participants by site and by discipline, showing individual site participation ranging from 11 to 18 individuals, and involving disciplines of occupational therapy, physiotherapy, occupational therapist and physiotherapist assistants, speech-language pathology, social work, and nursing.

**Table 3:** Participants who enrolled in CO-OP KT.

Discipline	Site 1	Site 2	Site 3	Site 4	Site 5	Total
Occupational Therapy	4 (30.8%)	4 (33.3%)	4 (36.4%)	7 (38.9%)	4 (36.4%)	23 (35.4%)
Physiotherapy	4 (30.8%)	2 (16.7%)	2 (18.2%)	5 (27.8%)	2 (18.2%)	15 (23.1%)
Occupational and Physical Therapy Assistant	3 (23.1%)	1 (8.3%)	1 (9.1%)	2 (11.1%)	2 (18.2%)	9 (13.8%)
Speech-Language Pathology	1 (7.7%)	1 (8.3%)	1 (9.1%)	2 (11.1%)	2 (18.2%)	7 (10.8%)
Social Work and Nursing	1 (7.7%)	4 (33.3%)	3 (27.3%)	2 (11.1%)	1 (9.1%)	11 (16.9%)
Total	13 (20%)	12 (18.5%)	11 (16.9%)	18 (27.7%)	11 (16.9%)	65 (100%)

Table 4 provides the mean and standard deviation scores for the MCQ and SERTA subscales at all time points. Table 5 provides the results of the mixed effects modeling for the MCQ and SERTA data. The effects of each time point are incremental from the previous, i.e. the value given for Intervention (T2 +T3) is the amount of change over Baseline (T1), and the value given for Follow Up (T4) is the amount of change over the Intervention value. The secondary model, shown in italics in Table 5, examined the relative effects of the workshop and the support period, and the values given reflect the approximate amounts of Intervention change that can be attributed to the Workshop (T2) or subsequent Support Period (T3). For the primary model, significant intervention effects were seen for two of the three SERTA subscales (CFT: Client-focused therapy and PCSU: Promoting cognitive strategy use) and the MCQ data, at T3 and T4., The secondary model, shown in italics revealed that the effect for the change in knowledge (MCQ data) came largely from the workshop. The post workshop change was 11.4 points, (6.3, 16.4) compared to -2.3 (-6.4,1,8) for the support period. For SERTA, the opposite was true, with the bulk of the effect coming from the support period and negligible change occurring following the workshop. For the most efficacious SERTA subscale, CFT, the effect of the workshop was 0.5 (-2.9, 3.9) and the effect of the support period was 4.7 (1.1, 8.3). Although not significant, there was a change in TDAT of 3.8 points from after the workshop to the end of the support period (T3), and an additional incremental change of 3.4 points from the end of T3 until the follow-up at T4.

**Table 4:** Means and standard deviations for MCQ and SERTA data at all time points.

Outcome	N	Site 1	Site 2	Site 3	Site 4	Site 5	Total
		**Multiple choice questions (MCQ)**					
Time 1	63	72.8 (10.4)	58.4 (18.8)	67.1 (13)	71.2 (9.4)	63.3 (14.1)	67.2 (13.7)
Time 2	52	83 (11.6)	80.4 (13.6)	72.2 (8.8)	83.4 (11)	81.7 (13.8)	80.5 (12)
Time 3	38	73.6 (10.5)	81.4 (6.6)	75 (13)	84.9 (9.5)	74.5 (12.7)	79.1 (11.1)
Time 4	35	78.8 (9.3)	79.1 (16.3)	69.2 (9.4)	79.8 (10.4)	73.8 (20.1)	77.1 (12.9)
		**SERTA–Top-down assessment and treatment (TDAT)**					
Time 1	65	59.6 (21.5)	68.8 (17.3)	60.4 (19.5)	65.1 (11.2)	64.1 (18.4)	63.7 (17.1)
Time 2	52	63.3 (9.8)	70.9 (15.7)	64.1 (16.1)	64.3 (8.3)	70.2 (16.7)	66.2 (13)
Time 3	39	60.5 (15.2)	73.4 (9)	64.3 (14.9)	69.2 (17.1)	76.2 (14.7)	68.6 (15.2)
Time 4	35	71.7 (5.1)	74.9 (20.9)	68.1 (17.7)	72.3 (9.8)	80.2 (13.4)	73.2 (13.5)
		**SERTA–Client focused treatment (CFT)**					
Time 1	65	67.1 (13.4)	64.9 (22.1)	61.3 (19.5)	61.1 (12.3)	68.6 (17.2)	64.3 (16.5)
Time 2	52	65.8 (11.9)	68.7 (17)	65 (18.5)	62.8 (12.4)	73.4 (15.8)	66.6 (14.8)
Time 3	39	70.9 (7.4)	77.7 (14.2)	65.2 (8.4)	63.2 (20.6)	78.3 (12.1)	69.4 (15.8)
Time 4	35	80.1 (9.9)	70.1 (15.8)	65.5 (21)	69.9 (11.6)	84.2 (11.9)	73.1 (14.5)
		**SERTA–Promoting cognitive strategy use (PCSU)**					
Time 1	65	63 (12.8)	64.4 (21.6)	62.5 (16.4)	64.4 (10)	63.4 (20.8)	63.6 (15.7)
Time 2	52	63.8 (12)	67.8 (15.7)	65.6 (14.5)	62.8 (11.1)	70 (16.4)	65.6 (13.4)
Time 3	39	71.3 (8.3)	79.2 (7.3)	60.8 (15.7)	65.5 (20.5)	77.5 (12.9)	69.8 (16.1)
Time 4	35	77 (8.6)	77.1 (13.3)	66.2 (16.2)	72 (8.1)	82.4 (10.5)	74.5 (11.5)

**Table 5:** Mixed effects models—Fixed effects [and 95% Confidence Intervals] and random effects in SERTA sub-scale domain scores and MCQ scores.

	Self-efficacy for top down approaches (SERTA)							
**Fixed Effects**	**CFT**	***p***	**PCSU**	***p***	**TDAT**	***p***	**MCQ**	***p***
Baseline (T1)	64.3 [60.4, 68.2]		63.6 [59.9, 67.4]		63.7 [59.8, 67.6]		67.1 [62.8, 71.4]	
Intervention (T2 +T3)	5.1 [1.6, 8.5]	0.005	5.4 [0.4, 10.4]	0.04	4.3 [-0.3, 9.0]	0.07	8.9 [5.2, 12.6]	<0.001
*Workshop (T2)*	*0*.*5* *[-2*.*9*, *3*.*9]*	*0*.*77*	*1*.*0* *[-2*.*2*, *4*.*1]*	*0*.*55*	*0*.*5* *[-3*.*9*, *4*.*9]*	*0*.*82*	*11*.*4* *[6*.*3*, *16*.*4]*	*<0*.*001*
*Support(T3)*	*4*.*7* *[1*.*1*, *8*.*3]*	*0*.*01*	*4*.*4* *[-0*.*5*, *9*.*4]*	*0*.*08*	*3*.*8* *[-0*.*8*, *8*.*4]*	*0*.*11*	*-2*.*3* *[-6*.*4*, *1*.*8]*	*0*.*28*
Follow Up (T4)	0.8 [-5.9, 7.5]	0.81	2.1 [-1.6, 5.8]	0.28	3.4 [-1.9, 8.7]	0.21	-1.9 [-6.9, 3.1]	0.46
**Random Effects (SD)**	**Participant Level**	**Site Level**	**Participant Level**	**Site Level**	**Participant Level**	**Site Level**	**Participant Level**	**Site Level**
Baseline	14.5	-	13.9	-	11.5	-	11.3	3.3
Intervention	5.6	-	7.5	3.9	-	-	5.0	2.1
Follow Up	-	6.4	-	1.8	-	-	-	4.3
Residual	7.0	-	6.6	-	11.1	-	6.4	-

Parameters in italics refer to secondary models in which an additional time point, the post-workshop assessment, was included in the analysis. Because not all therapists completed both T2 and T3 assessments, the figures from the secondary model do not exactly sum to the combined (T2+T3) estimate from the primary model. Intervention refers to CO-OP KT as a whole, including the workshop and the implementation support period.

The medical record audit examined eight charts per site, 40 in total, 6 months before CO-OP KT and another 40 in the month following CO-OP KT. No charts showed any evidence of CO-OP use at baseline, compared to 8/40 (20%) charts from four out of five sites following CO-OP KT. Evidence of client-centred goal-setting was present in 29/40 (72.5%) charts at baseline and 34/40 (85%) charts after the KT intervention (chi-square 1.86, p = .17). The total number of functional goals set for all sites before and after CO-OP KT was 185 and 198, respectively; the number of impairment-based goals set for all sites before and after CO-OP KT was 92 and 77, respectively (a reduction of 6%); and the number of component-based goals set for all sites before and after CO-OP KT was 9 and 25, respectively (an increase of 7%). There was an association between goal type and assessment time (chi-square = 8.973; p = .011). Post-hoc test revealed that there were more component goals in the post group (z = 2.7, p = .007).

## Discussion

The CO-OP KT intervention was associated with significant improvements in knowledge, aspects of self-efficacy, and aspects of practice related to the multi-site implementation of the CO-OP Approach in inter-professional stroke rehabilitation teams. Knowledge, self-efficacy in promoting cognitive strategy use and self-efficacy in client-focused therapy were all maintained 6-months after the CO-OP KT intervention ended. There was no significant change in self-efficacy for using a top-down approach. Changes in knowledge occurred after the workshop and were largely maintained but not augmented during the support period, whereas changes in self-efficacy occurred predominantly during the support period rather than after the workshop. The audit of medical records revealed some limited evidence of practice change. In this discussion, we suggest mechanisms that may have influenced the improvements in knowledge and self-efficacy, suggest potential reasons why change did not occur in self-efficacy for top-down approaches, expand on the findings from the audit of medical records, and outline study limitations and future directions.

The improvements demonstrated in knowledge and aspects of self-efficacy represent important shifts towards a implementing a collaborative person-provider rehabilitation model, and we postulate these successes come from the strong and embedded researcher/knowledge user partnership, a focus on site-specific implementation goals and site-driven implementation strategies, and the use of existing health system (stroke network) education and communication infrastructure. Furthermore, we suggest that these aspects of the intervention are likely generalizable to other KT projects that aim to shift the care dynamic towards a more equal person-provider partnership. Scholl et al’s scoping review identifies the importance of organizational and system-level characteristics in implementing shared decision making-based approaches into routine care [46]. The maintenance of improvements may have been facilitated by the individual teams’ ownership of implementation goals and strategies and the comprehensive sustainability plan that was implemented after the formal implementation support period.

No significant change occurred in health care providers’ self-efficacy related to top-down approaches. It may be that this is a theoretical construct that does not have obvious practical applicability for providers and was thus not integrated into their clinical reasoning. It may also be that a bottom-up, impairment focus is so deeply ingrained in the larger stroke rehabilitation system that making this shift will require system-level changes before teams and individuals can adopt this thinking. However, there are a number of important barriers to such a shift. First, the Canadian Stroke Best Practice Guidelines, which guide practice in Ontario, implicitly define stroke rehabilitation as a process of expert-led impairment reduction [23]. Improved adoption of promising top-down interventions that focus on person-centred goals may not be possible without explicit examination of underlying ideas about what stroke recovery is and how rehabilitation institutions that provide stroke recovery care can best support it. Second, bottom-up, neurophysiological approaches such as Bobath [47] or Neurodevelopmental Treatment [48], which lack evidence of effectiveness [20], are still widely used in stroke rehabilitation. A Cochrane Collaborative review reported a significant detrimental effect of these neurophysiological types of approaches on independence in activities of daily living and gait velocity [20], suggesting that these approaches require de-implementation. Presad and Ionnadis (2014) [49] have said that de-implementation may meet “fierce tactical resistance”, and that evidence wars, in which providers evoke outdated or lesser-quality evidence, can hinder de-implementation. De-implementation is largely understudied in implementation science [50], but evidence is emerging to suggest that the barriers and facilitators are different than those related to implementation and may need to be addressed separately [51].

Our audit of the medical records provided some concrete evidence of practice change, in that no records mentioned aspects of CO-OP use prior to the KT intervention and 20% mentioned it after. However, in real terms, only eight of 40 charts audited post intervention had any documentation related to use of the CO-OP Approach, and one site showed no evidence at all. Although this is likely evidence of incomplete implementation of CO-OP, it was also probably compounded by the lack of relevant documentation structures. During the implementation support period, all teams asked the implementation facilitators for advice on documentation. We had not developed documentation structures, because we believed that sites would develop their own procedures that fit within their existing site-specific systems. In hindsight, the time and energy required to develop documentation procedures might place on front-line providers, in addition to learning and implementing a new technique that includes new terminology, was too burdensome. In future similar projects, we recommend providing a documentation framework or guidelines as a starting point that teams can then modify to suit their context.

### Study limitations

This study was limited in that it was a single arm study with no control group, occurring in a large and complex health system over several months, in which many potentially confounding events may have occurred. Thus, it is impossible to know with certainty whether changes seen were entirely due to the CO-OP KT intervention, or whether other system factors may have had influence. Additionally, multiple statistical comparisons may have overestimated some of the positive findings. As the CO-OP KT intervention was multi-faceted, and was studied as a whole, it is difficult to know which components were more or less effective than others. Not providing a documentation framework for participants may have hindered both their CO-OP implementation and our ability to use medical record audit as an indicator of practice change. While it is likely that the large degree of control given to individual sites for implementation goals and plans may have contributed to the overall success of the project, the issues with documentation highlight the importance of having individualization occur within a clear, structured framework.

## Conclusion

CO-OP is a complex clinical intervention in which clients are taught to use problem-solving cognitive strategies to acquire personally-meaningful functional skills, and health care providers are required to shift control regarding treatment goals and intervention strategies to their clients. In using an inter-professional multi-faceted KT intervention in which similar principles were applied (i.e. control over implementation goals and strategies was shifted from researchers to the participating teams), documented practice change was present but limited, and sustained improvements in health care providers’ knowledge and aspects of self-efficacy.

## Supporting information

S1 File Study protocol. (PDF)

S1 Checklist TREND statement checklist. (PDF)
