# Supplementary material for: Implementing a function-based cognitive strategy intervention within inter-professional stroke rehabilitation teams: Changes in provider knowledge, self-efficacy and practice
Source: PLoS One. 2019 Mar 11;14(3):e0212988. doi: 10.1371/journal.pone.0212988 (PMC6411258; doi:10.1371/journal.pone.0212988)
Supplement: S1 File — (PDF) [file pone.0212988.s001.pdf]

Building Capacity in the System to Support Persons with Stroke and Cognitive Impairment: An Evidence-based, Multi-faceted, Knowledge Translation Approach

Sara McEwen[1] (Co-PI), Elizabeth Linkewich[2] (Co-PI), Michelle Donald[2], Deirdre Dawson[3], Mary Egan[4], Anne Hunt[5], Sylvia Quant[2], Sharron Runions[2]

[1]Sunnybrook Research Institute, [2]Sunnybrook Health Sciences Centre, [3]Rotman Research Institute, [4]University of Ottawa, [5]Bloorview Research Institute

1.0 Introduction

Patients with cognitive impairments following a stroke are often *denied access to inpatient rehabilitation*,[1] despite evidence of its benefits for them.[2] These patients comprise up to 30% of stroke patients.[3] In the Toronto Stroke Networks (TSNs), the few patients with cognitive impairment admitted to inpatient stroke rehabilitation generally receive services based on outdated impairment-reduction models, rather than recommended function-based approaches.[4] These two issues, reduced access to rehabilitation and the knowledge-to-practice gap, both stem from a reported lack of skills and knowledge on the part of stroke rehabilitation teams to foster recovery in people with cognitive impairments.[4] To address these issues, we will implement and evaluate a multi-faceted, supported, integrated knowledge translation (KT) initiative, targeted specifically at the inter-professional application of the Cognitive Orientation to daily Occupational Performance (CO-OP),[5] called CO-OP KT. CO-OP is a contemporary, effective, cognitive strategy-based treatment approach aligned with Canadian Stroke Best Practice Recommendations for cognitive rehabilitation.[6]

Lead knowledge user and decision maker, Ms. Linkewich, is a regional director in the TSNs. The TSNs consists of 3 of Ontario's 11 regional stroke networks devoted to regional implementation of evidence-based practice to improve outcomes for people with stroke.[7] Ms Linkewich has convened a knowledge user team of educators, a community engagement specialist, and management collaborators. They have been involved with every aspect of this project's design, beginning with identifying the problem and developing a plan to address it. Further, they have a track record of conducting and disseminating health system research,[1, 8-13] and have integrated with a group of researchers, led by Dr. McEwen, who have expertise in the development and evaluation of complex interventions, cognitive impairment, health professional behaviour, and KT.

The Knowledge to Action (KTA) framework developed by Graham et al provides the foundation for this project.[14] It consists of a central knowledge creation cycle and a concurrent action cycle. Figure 1 depicts the KTA framework with content specific to the CO-OP KT project. In preparatory work, we conducted the first of the action phases, identifying issues and synthesizing knowledge to mitigate those issues. This project begins with the phase of adapting the knowledge to the local context and identifying barriers to knowledge uptake, and will continue with selecting, tailoring, and implementing KT intervention, monitoring and sustaining knowledge use and evaluating outcomes. Also informing the project are the inter-professional care (IPC) and inter-professional education (IPE) constructs. IPC promotes effective working relationships among health care providers from different disciplines and their patients and enables optimal health outcomes by building on the foundational elements of "respect, trust, shared decision making and partnerships".[15] IPE emphasizes providers from multiple differences learning with, from, and about each other, and is interdependent with IPC.

This project will advance knowledge about the degree to which the implementation of a supported, integrated, inter-professional KT initiative can sustainably change health system outcomes (access to rehabilitation), knowledge outcomes (rehabilitation team practice), and patient outcomes (functional improvement). These changes will be sustained over the long-term through integrated KT mechanisms and partnerships with health and academic institutions.

The long-term objective of CO-OP KT is to optimize functional outcomes for individuals with stroke and cognitive impairments. Three outputs are expected:

1. Increased proportion of patients with cognitive impairments admitted to inpatient stroke rehabilitation;

2. Enhanced capacity of inter-professional team members to implement a cognitive-strategy based treatment approach;
3. Improved immediate and long-term functional outcomes for patients with cognitive impairments discharged from inpatient stroke rehabilitation.

The TSNs have developed a model to aid discharge from acute stroke care in a way that maximizes both patient outcomes and the efficient use of scarce rehabilitation resources, dependent on stroke severity and patient characteristics.[1] The model predicts 40% of patients discharged from acute care should be transferred to specialized inpatient rehabilitation. In practice, the proportion is only 26%. One prevalent and modifiable reason for this discrepancy is that patients classified with severe stroke, often with cognitive impairments but meeting eligibility criteria, frequently have their inpatient rehabilitation referrals declined. The TSNs estimate that admitting this group to inpatient rehabilitation would *give access to an additional 286 patients* in Toronto each year.[1] Lack of access to inpatient rehabilitation may result in patients being transferred to assisted living facilities without the benefits of rehabilitation that could potentially have enabled them to return home. It may also contribute to the relatively poorer outcomes of patients with cognitive impairments, such as higher rates of dependency and disability and lower mood and quality of life than those without.[16]

Interviews with clinicians from 5 stroke rehabilitation teams were conducted to determine the reasons that admissions for patients with cognitive impairments are frequently declined.[4] Clinicians report they deny admission to these patients because they do not have the specialized knowledge and skills necessary to facilitate their functional recovery. The interviews also revealed the rehabilitation treatments used with the few patients with cognitive impairments admitted to rehabilitation were based on outdated impairment-reduction models, rather than on currently recommended function-based models that incorporate problem solving and strategy training. The contemporary models are substantially better than pure impairment reduction models, in that they alone are associated with sustained improvements in real-world functioning, self-evaluation, and problem solving, among several other benefits.[2]

Canadian Stroke Best Practice Recommendations state that interventions for patients with cognitive impairments should be tailored to meet meaningful, functional patient goals.[6] CO-OP aligns with these recommendations, and has demonstrated efficacy to improve function in people with stroke [17-19], including those with demonstrated cognitive impairment [19-21]. CO-OP has an established training infrastructure with available local trainers, and therefore is feasible and efficient to implement across the TSNs. However, given the multi-institutional environment and the need for shifts in attitudes and beliefs, and the relative ineffectiveness of passive KT strategies,[22] CO-OP training by itself is unlikely to be sufficient to cause widespread, sustained practice change. Thus, we propose to implement a multi-faceted KT initiative that will combine institution-specific support, multi-sectoral collaboration, and managerial participation along with the established CO-OP training. These additional health system components are important in moving evidence to practice in complex environments, particularly when shifts in culture, attitudes, and behaviour are required.[23, 24] CO-OP KT will also integrate seamlessly with existing TSN education and KT infrastructure (See Figure 2), and will incorporate online components; these aspects will ensure sustainability.

Although Ontario's Ministry of Health and Long-Term Care and the TSNs have embedded access to inpatient rehabilitation for patients with cognitive impairments into care expectations,[25] the inter-professional teams' perceived lack of skills to support patients with cognitive impairment remains a

major barrier. Within the TSNs, stated reasons for declining admission to rehabilitation have included, "*the patient would benefit from a rehab program with a strong cognitive component,*" or a similar comment, implying the team cannot address their needs. Since no such specialized cognitive rehabilitation programs exist, the patient may not get rehabilitation services at all. Through CO-OP KT, we expect to see improved knowledge and practice change in inter-professional stroke rehabilitation teams, manifested as greater implementation of cognitive rehabilitation best practices and greater self-efficacy to do so. Following directly from positive practice change, we also expect to see improvements at the health system and health outcome levels. Individual rehabilitation professionals and teams as a whole will come to see themselves as having the specialized knowledge and skills to facilitate functional improvement in patients with cognitive impairments. With this, the number of patients with cognitive impairment who are accepted to inpatient rehabilitation will increase. Further, because previous research indicates that CO-OP is associated with reductions in disability and improvements in activity performance, self-efficacy, and participation, including in those with demonstrated cognitive impairment,[17-21, 26-28] we expect to see similar positive patient outcomes.

2.0 Methods

2.1 Questions

Three specific research questions are posed, aligned with the 3 expected project outputs:

1. Is the implementation of CO-OP KT associated with a change in the proportion of patients with cognitive impairment accepted to inpatient stroke rehabilitation?
2. Is the implementation of CO-OP KT associated with a change in rehabilitation clinicians' practice and self-efficacy related to implementing this evidence-based stroke rehabilitation approach, immediately following and 1 year later?
3. Is CO-OP KT associated with changes in activity, participation, and self-efficacy to perform daily activities in patients with cognitive impairment following stroke at discharge from inpatient rehabilitation and at 1, 3, and 6-month follow-ups?

2.2 Study Design and Procedures

To answer these 3 questions, 3 interrelated studies will be conducted. *See Figure 3 for a visual depiction of all 3 studies.* The first, which relates to changes at the health system level, will be addressed using a quasi-experimental, interrupted time series design (Study 1); the second, which relates to changes in health care professional practice and self-efficacy, will be addressed using a single group pre-post evaluation design (Study 2); and the third, which relates to patient outcomes, will be addressed using a non-randomized design with historical controls (Study 3).

2.2.1 Study 1

An interrupted time series (ITS) design provides an estimate of the effect of an intervention using a long series of measurements of dependent variables, divided into pre-intervention and post-intervention segments.[29] It is useful when randomized designs are impossible or impractical, as is the case here.

Monthly summaries of stroke unit level data are available retrospectively from 2012 onwards. Monthly ITS pre-intervention time points will begin with retrospective data for 18 months prior to the study start date, and will continue every month for an additional 10 months after the study start date. Thus, there will be 28 pre-intervention time points, called –T28 (intervention implementation minus 28 months), -T27, etc, to –T1. Following –T1, the CO-OP KT intervention will occur, and will take 3 months to complete. The intervention time point is called T0, and no data collection will occur. Next,

15 post-intervention monthly time points called T1, T2, etc, to T15 will occur. Aggregate electronic data will be collected at all time points from the National Rehabilitation Reporting System (NRS) and the TSN's E-Stroke Rehab Referral System. Data elements (fully described in Table 1) will include monthly summaries of rehabilitation referrals, rehabilitation admissions, referrals declined, reasons for declined referrals, age, sex, Functional Independence Measure (FIM)™[30] motor and cognitive scores, and discharge location.

2.2.2 Study 2

A pre-post study with a single group of stroke rehabilitation clinicians will be conducted. All stroke rehabilitation clinicians from the 5 sites will be invited to participate, and consenting staff will be enrolled. Outcome measures are fully described in Table 1. Chart audits will be used to assess practice change,[31] and a self-report survey will evaluated changes in self-efficacy; these will be administered immediately before and after the CO-OP KT intervention and at a one-year follow-up. To develop an in-depth understanding of team processes, practices, attitudes related to adoption and sustainability of best practices for cognitive rehabilitation, site-specific focus groups will be held with consenting clinicians before the intervention and at the one-year follow-up.

2.2.3 Study 3

To answer Question 3, a non-randomized study using historical controls will be conducted. We will compare a group of patients treated by stroke teams who have been exposed to the CO-OP KT intervention (Intervention) to a group who have not been exposed (Historical Control). Prior to the implementation of CO-OP KT, control participants will be recruited, and after the CO-OP KT intervention, intervention participants will be recruited. During the 3-month period when the CO-OP KT intervention is being administered to teams and learning is being consolidated, no recruitment will occur. In addition to data elements universally collected for the ITS Study 1, Study 3 participants will undergo additional assessments to measure activity performance, participation, and self-efficacy. Data collection points will occur at the individual patient participants' admission to rehabilitation, discharge from rehabilitation, and at 1, 3, and 6 months following discharge. Follow-up assessments will be administered by telephone, a feasible and cost-effective alternative to face-to-face assessments.[32] Data will be collected by family member proxy if the patient participant is not able to do telephone interview.[33] Table 1 provides a description of all outcomes, indicators, and timing of administration.

2.3 Recruitment and Sample Sizes

All 5 inpatient stroke rehabilitation units or combined stroke/neurology units in the TSN have agreed to participate and have provided letters of collaboration. Based on TSN data, we estimate that the 5 units together will have approximately 80 admissions per month combined to contribute to the aggregate monthly data. For Study 1, we will collect data from all patients aged 18 years or more who have completed inpatient rehabilitation with a primary diagnosis of stroke, defined as Rehabilitation Client Group (RCG) 01.1, 01.2, 01.3, 01.4 or 01.9. For Study 3, a subset of those patients will be recruited and the additional criteria of having at least some cognitive impairment will be applied, determined using the Montreal Cognitive Assessment (MoCA).[34] The MoCA is a 30-item test of cognitive impairment that includes elements of short-term memory recall; visuospatial capacity; aspects of executive functioning; attention, concentration, and working memory; language; and orientation. Patients with scores lower than 26 will be included. Those with scores of 26 and higher are less likely to have cognitive impairment. Additionally, patients will be required to have sufficient language skills to complete the additional assessments described in Table 1. Exclusion criteria are

neurological diagnoses other than stroke, the presence of major psychiatric illness or have capacity issues requiring the use of a substitute decision maker under Ontario's Substitute Decision Maker Act. Based on data from a published study,[21] a sample size of 13 per group will have 80% power to detect a between-group difference of 9 points on the FIM™, standard deviation of 8. Allowing for 30% attrition from all sources, we will recruit 17 participants per group, 34 in total. Based on past experience, we expect a consent rate of 1 participant per site per month. Thus, recruitment is highly feasible, and will likely be completed in 4 months for each group.

For Study 2, all clinicians will be invited to participate, and those who provide informed consent will be included in the survey and focus groups. The 5 stroke teams have approximately 50 staff each, or approximately 250 total. Based on past experience, we expect a participation rate of 50% for the self-efficacy survey and about 30% for the focus groups. Therefore, we anticipate running 2 focus groups with 7-8 participants each at each of the 5 sites, for a total of 10 focus groups per assessment period, and 20 focus groups total. Chart audits will be conducted on 80 charts of patients discharged approximately 6 months (+/- 1 month) before CO-OP KT implementation, and another 80 charts at both 6 and 12 months (+/- 1 month) after CO-OP KT implementation, providing 89% power to determine a 25% change in the audit criteria outlined in Table 1.[35]

2.4 Data Analysis

Descriptive statistics will be compiled for all quantitative data collected. The ITS (Study 1) will be analyzed using simulation modeling analysis,[36] a trend analysis that provides good power to detect post-intervention trend changes when analyzing 30 time points or less per segment. As a precursor to modeling, the time series data will be plotted, analyzed visually for trends, and the degree of autocorrelation will be calculated. For Study 2, pre/post changes on the clinician self-efficacy survey will be calculated using the paired t-test, assuming the data are normally distributed or the signed rank test if not. Qualitative focus group data will be analyzed using directed content analysis,[37] with initial codes based on questions derived from the focus group questions. For Study 3, the non-randomized trial of individual patients with historical controls, between- and within-group differences on the outcome measures will be examined using repeated measures ANOVA.

2.5 Description of the CO-OP Treatment Approach

CO-OP, a functional, patient-goal-centred, problem solving approach, is associated with improved function, activity performance, participation, and self-efficacy in people with stroke,[17, 18, 20] and has demonstrated better efficacy than control treatments.[19, 21, 28] CO-OP has 7 key features that include client-chosen goals, dynamic performance analysis, cognitive strategy use, and guided discovery. In the first meeting, the patient and a rehabilitation clinician work together, using the Canadian Occupational Performance Measure (COPM),[38] to select personally-important activities, which then form the basis of their rehabilitation goals and become the focus of the intervention. In the second meeting, the patient is taught a global cognitive strategy (GOAL-PLAN-DO-CHECK). In all subsequent sessions this strategy is used in an iterative manner as the main problem-solving framework to facilitate skill acquisition/goal attainment. The patient identifies a GOAL, and then is guided by the clinician to discover a PLAN to achieve the goal. The patient is then asked to DO the plan, and subsequently to CHECK to see if the plan was implemented and if it worked, i.e. the goal was achieved. Within the PLAN phase, the clinician uses guided discovery rather than explicit instruction to help the patient analyze the task to be performed and to discover domain-specific strategies that are specific to the particular performance problems of that patient with that activity. A detailed description

of CO-OP's theoretical foundations, key features, and administration procedures is available in a publication by Polatajko and Mandich, 2004.[5]

2.6 Description of the CO-OP KT Intervention

CO-OP KT consists of CO-OP training for the inter-professional team and subsequent implementation support (KT). Two levels of CO-OP training will be provided: 1) A 1 to 2 hour Introduction to CO-OP, using the Project Partner University of Toronto's *Introductory CO-OP e-Learning Module* and 2) Advanced training in the form of a 2.5 day hands-on *CO-OP Workshop*. Team members (e.g. occupational therapists, physiotherapists, speech-language pathologists, nurses in certain roles, etc.) who are directly involved with teaching each skills for which CO-OP is effective, such as mobility, activities of daily living, or communication, will receive both components. Team members who are not directly involved in teaching functional skills (e.g., physicians, most nurses, dieticians etc.) will be introduced to CO-OP using the first introductory component so that they can support and encourage the use of the approach. Funds have been budgeted to cover clinician time when they are participating in the either level of training.

KT support will be provided to facilitate implementation and sustainability of the CO-OP approach. This support uses evidence-based behavior change strategies and will be delivered by an Implementation Facilitator, to be employed by the project. We will recruit an Implementation Facilitator who is a health care professional with experience in both stroke rehabilitation and in implementing educational and knowledge translation initiatives. He or she will make linkages between CO-OP content and existing TSN KT infrastructure. See Figure 2 for a detailed visual depiction of existing TSN KT infrastructure. The Implementation Facilitator will work closely with project knowledge users Michelle Donald (Regional Education Coordinator), Sylvia Quant (Regional Rehab & Community Re-engagement Coordinator), and Sharron Runions (Clinical Nurse Specialist). Content will be tailored for the unique environment of each individual team, and the Implementation Facilitator will be available throughout the project for consultation by telephone or email, will moderate a CO-OP discussion forum on the TSN's online Virtual Community of Practice (VCoP) (www.strokecommunity.ca), and will be onsite at each participating institution regularly throughout the project for one-on-one face-to-face coaching and issue resolution. The Implementation Facilitator will also consult with Sunnybrook Health Sciences Centre (SHSC) Inter-professional Care (IPC) Advisory Committee, to ensure CO-OP KT incorporates key IPC competencies (see letter of collaboration).

2.7 Sustainability

Both the sustainability of training and the sustainability of knowledge uptake have been considered. Our partnership with Continuing Education, Department of Occupational Science and Occupational Therapy (OS&OT) at the University of Toronto supports the sustainability of CO-OP training, in that the Introductory CO-OP e-Learning Module will be available and updated on a long-term basis. Additionally, the stroke network will receive preferred access to an online CO-OP workshop module. This module, currently under development by members of the Department of OS&OT and intended to be an alternative to current face-to-face CO-OP workshops, will provide clinicians who join the inter-professional teams after the CO-OP KT implementation with a means of receiving advanced CO-OP training in a timely and cost-effective manner.

Sustainability of uptake will be ensured by TSN KT infrastructure currently in place (see Figure 3), as well as through sustainability processes developed locally at each site, in collaboration with the Implementation Facilitator.

2.8 Potential Challenges and their Solutions

An issue inherent to time series studies is the possibility that changes not related to the intervention will occur within the system and impact the outcome of interest. By collecting pre-intervention data for 28 months, including 18 months of retrospective data, these trends will be apparent and can be taken into account during analysis. Additionally, time series data will be examined in two ways: we will examine all 5 units as a group, and also as individual cases. By examining the trends of individual units, any outcome-impacting changes within a single unit, such as a sudden staffing shortage, can be detected and explained.

Influencing practice changes among an entire health system is expected to be challenging. In addition to addressing knowledge, skills, and attitudes of individual clinicians, institutional culture and support for practice change will be significant factors. The Implementation Facilitator will work together with teams to develop local, site-specific content to help mitigate some of these concerns. Two pragmatic barriers to evidence uptake by health care professionals are lack of time and lack of resources. We have budgeted for clinician time to attend workshops. Additionally, we have included the position of Implementation Facilitator to provide the teams with a human resource with the time and ability to develop materials on their behalf and to provide support while new knowledge and skills are being adopted and consolidated. Use of the VCoP across sites also provides just-in-time access to peers and experts to support ongoing learning and implementation needs. Access to rehabilitation for persons with cognitive impairment has been identified as a priority by the TSN Acute and Rehab Stroke Flow Working Groups, with representation from decision-makers across Toronto organizations. These decision-makers have made a collective commitment to local implementation and will support this project across the system.

Patient recruitment among the stroke patient population is challenging.[39] To mitigate this and reduce the burden on point of care clinicians, our inclusion criteria are broad and we have requested budget for dedicated research assistant time for recruitment and retention.

Rehabilitation team member adoption of the CO-OP approach may be an issue. Intervention fidelity will be monitored by the Implementation Facilitator, who will observe at least 1 video recorded treatment session from each team member who has taken the advanced CO-OP workshops and rate them using an existing CO-OP treatment fidelity checklist. This will also act as a feedback and instructional mechanism. As an additional means to monitor adoption, regular reporting on the local status of CO-OP implementation will occur to TSN decision makers through existing channels.

As we are working with a large project team and 5 separate sites, communication may be a challenge. All project team members are experienced in working collaboratively with large groups. We will make use of existing infrastructure to adhere to timelines, budgets, and outputs, and will have regularly pre-scheduled teleconferences for monitoring those and addressing emerging project issues in a timely manner.

2.9 Timeline

Figure 3 includes a visual depiction of the timeline. Prior to the project beginning, approval will be sought from all relevant ethics review boards, and retrospective stroke unit level data will be aggregated. The first 10 months of Year 1 will be the pre-intervention phase, in which unit level data collection will continue (Study 1), and recruitment of patient controls (Study 2) and health care

professionals (Study 3) will occur. Towards the end of Year 1 and beginning of Year 2 (months 11-13), the CO-OP KT intervention will occur. The remainder of Year 2 and the first half of Year 3 will be the post-intervention phase, in which unit level data collection continues (Study 1), recruitment of the patient intervention group (Study 2) occurs and follow-up data collection occurs with consenting health care professionals (Study 3). The final half of Year 3 will be used for data analysis and report preparation.

3.0 Expertise and Resources

Dr. McEwen and Ms. Linkewich will co-lead the project. Dr. McEwen has a physiotherapy background, and 2 decades of experience in stroke research, including leading multi-site, multi-year projects. She adapted the CO-OP treatment approach for stroke,[17, 18, 40] and recently led a randomized controlled trial, comparing CO-OP to standard rehabilitation in people with subacute stroke.[19] She will devote 7.5 hours per week (0.2 FTE) to the project.

Ms. Linkewich has a background in occupational therapy and is the Regional Director for the North and East GTA Stroke Network (NEGTASN), based at SHSC. She works closely with the other Toronto-based stroke networks, meeting regularly with system decision-makers in Toronto and across Ontario. She has led large-scale health system change in the Toronto area. She chairs several provincial committees, including the Ontario Stroke Network's Knowledge Translation and Accountability subcommittee for stroke evaluation. She will devote 3.5 hours per week (0.1FTE) to the project.

Ms. Michelle Donald is the Regional Education Coordinator for the NEGTASN, and has clinical experience with cognitive impairment and a background in developing educational initiatives. Ms. Donald will be responsible for coordinating the implementation plan and will work closely with the Implementation Facilitator to support inter-professional teams, including facilitating use of the VCoP as a resource. Ms. Donald will commit 7.5 hours per week (0.2FTE) to this project.

Drs. Dawson, Egan, and Hunt are research project experts with backgrounds in stroke, occupational therapy, cognitive rehabilitation, health professional behaviour, and KT. Dr. Dawson will consult on cognitive rehabilitation and will oversee the CO-OP training. Dr. Hunt will conduct focus groups with inter-professional team members and provide consultation and support to the Implementation Facilitator regarding facilitating attitudinal and behavioural practice shifts. Dr. Egan will consult on study design and implementation and evaluation of integrated KT. Each will commit 1 hour per week to the project.

Dr. Quant and Ms Runions are knowledge users within NEGTASN with backgrounds in physiotherapy and nursing respectively. Dr. Quant is the Rehab & Community Re-Engagement Coordinator and will ensure CO-OP KT addresses long-term community re-engagement, committing 3.75 hours per week (0.1FTE). Ms. Runions is a clinical nurse specialist and the Chair of the TSN GTA Stroke Nursing Leaders Committee. Ms. Runions will consult on development and implementation of aspects of the approach pertaining to nursing, committing 2 hours per week (0.05 FTE).

The resources and environment available to ensure the success of this project are exceptional. Project partner NEGTASN within SHSC is highly committed to completing the project, as it is aligned with the stroke network's strategic priority to *implement high quality coordinated stroke care*, and is aligned with SHSC's values of excellence, collaboration, accountability, respect and engagement. The

TSNs existing KT infrastructure (Figure 3) is complex, supported with human and financial resources and well-positioned to absorb CO-OP KT. Sunnybrook Research Institute, where Dr. McEwen has her primary appointment, provides the research infrastructure to support this multi-site, multi-year project. Additional project partner, the Department of OS&OT at the University of Toronto, permits access to ongoing online CO-OP training, thereby ensuring not only sustainability but also that the content continues to be at the leading edge internationally.

Table 1: Outcomes, Indicators, and Timing for All Studies

Outcomes	Indicators and Description	Timing
Study 1: Health System: Data obtained from electronic referral system, health record, and NRS		
Access to inpatient rehabilitation	Monthly totals: # of inpatient rehab referrals, # of admissions, # declined; Reasons for declined referrals	-T28 to –T1<br>T1 to T15
Inpatient rehab outcomes	Average monthly Functional Independence Measure (FIM™) motor and cognitive scores (admission, discharge, and change)<br>Monthly frequency of discharge locations (home, home with services, assisted living facility, or acute care)	-T28 to –T1<br>T1 to T15
Study 2: Health Knowledge: Data obtained from stroke rehabilitation team members and chart audits		
Rehabilitation team member practice change	Chart audits will be conducted 6 months (+/- 1 month) before CO-OP KT implementation as a baseline and to confirm practice gaps previously identified with interviews,[4] and repeated at 6 and 12 months (+/- 1 month) following the CO-OP KT intervention. The chart audit review criteria will centre around documentation of functional goals (e.g. independence with upper body dressing), rather than impairment-reduction goals (e.g. increase arm strength); evidence of patient involvement in the goal-setting process; evidence of teaching of cognitive and problem-solving strategies as an intervention technique; evidence of use of guided discovery as an intervention technique.	-T6<br>T6, T12
Stroke rehab professional self-efficacy with knowledge and skills related to CO-OP	CO-OP Essential Elements Self Efficacy Tool: Participants are asked to rate their ability to perform 25 elements on a 10-point scale, with 1 indicating that they cannot perform the element at all and 10 indicating that their performance is excellent. Face validity evaluated by five members of the International CO-OP Academy.	-T1<br>T1<br>T12
Team perceptions and experiences with team processes, practices, attitudes related to adoption and sustainability of best practices for cognitive rehabilitation	Semi-structured site-specific focus group with groups of 5-8 team members at a time. Focus groups will be conducted by experienced facilitator Dr. Anne Hunt who will begin with an open-ended question “What has been your experience with facilitating recovery in patients with cognitive impairment?” Based on responses, Dr. Hunt will probe to obtain a thorough understanding of perceptions and experiences from a wide range of team members at each site.	-T1<br>T12
Study 3: Health Outcomes: Data obtained from consenting individual patients		
Performance on personally-meaningful, self-selected activities	The Canadian Occupational Performance Measure (COPM) is a standardized instrument for eliciting performance issues from the client perspective, and for capturing perceived changes in performance over time.[41] The COPM has demonstrated test-retest reliability of 0.89 in people with stroke.[42] A change of 2 points or more on the COPM is considered clinically significant.[41]	Admission to inpatient rehabilitation<br>Discharge from inpatient rehabilitation<br>1 month post discharge<br>3 months post discharge<br>6 months post discharge
Self-efficacy to perform daily activities	The Self-Efficacy Gauge (SEG) was designed to measure an individual’s self-efficacy in his or her ability to perform daily occupations that span a range of self-care, productivity, and leisure activities. Participants are asked to rate their confidence in their ability to perform 28 items, each on a 10-point scale, with 1 representing “not confident at all” and 10 representing “completely confident”. The SEG has very high internal consistency (0.94) and test-retest reliability (0.90).[43]	
Health status	The Stroke Impact Scale (SIS)[44] is a 59-item questionnaire about the perceived impact of stroke on function and everyday life. The SIS evaluates eight domains. Each item is scored on a 5-point Likert scale related to the degree of difficulty the person with stroke is experiencing. The SIS is widely used in stroke intervention studies as an outcome measure and the psychometric properties of the instrument are well-defined.[44-46]	
Cognitive screening tool	The MoCA is a 30-item test of cognitive impairment that includes elements of short-term memory recall; visuospatial capacity; aspects of executive functioning; attention, concentration, and working memory; language; and orientation. The MoCA has an excellent internal consistency (0.83) and test-retest reliability (0.92).[34]	

Note. CO-OP=Cognitive Orientation to daily Occupational Performance; KT=knowledge transfer

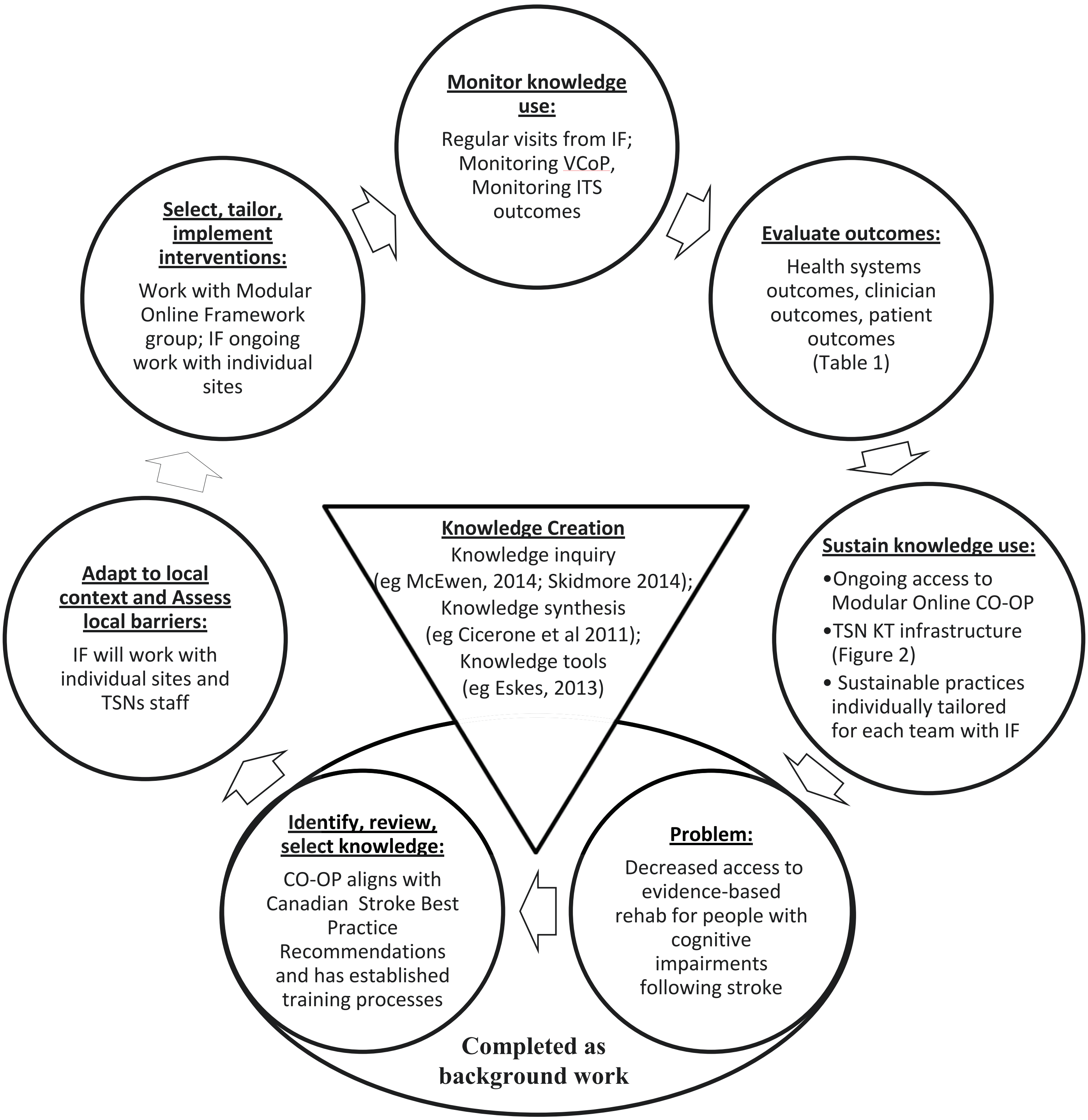

Figure 1: Knowledge to Action Framework with CO-OP KT Project Content. Adapted from Graham et al. (2006), the KTA Framework consists of an inner knowledge creation cycle, depicted here as an inverse triangle, and a concurrent action cycle, depicted here as the external circles. CO-OP=Cognitive Orientation to Occupational Performance; IF=Implementation Facilitator; TSNs=Toronto Stroke Networks; VCoP=Virtual Community of Practice; ITS=Interrupted Time Series; KT=Knowledge translation.

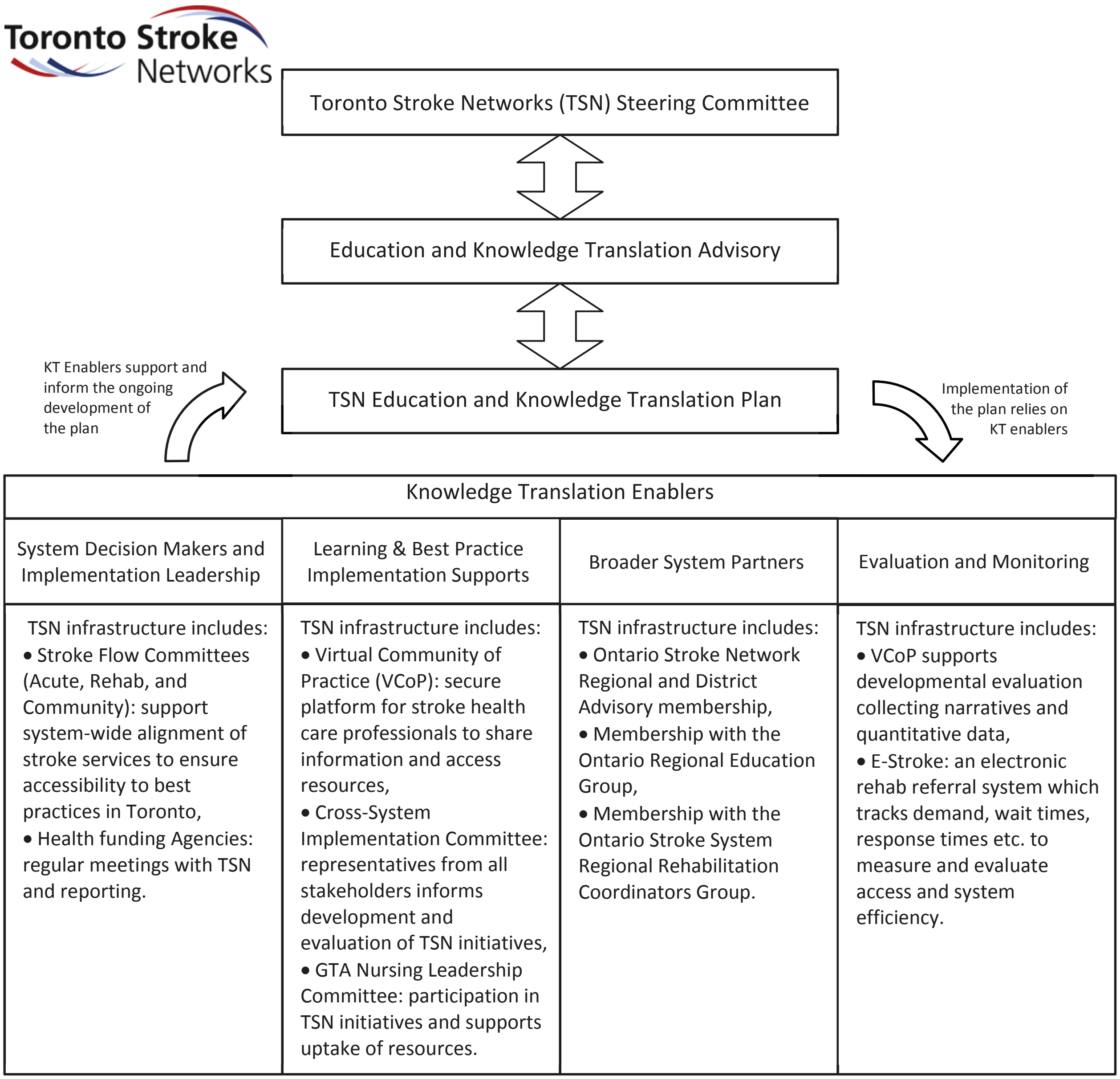

Figure 2: Toronto Stroke Networks Knowledge Translation (KT) Infrastructure.

Months	*Pre Study*	1-3	4-6	7-10	11-13	14-15	16-18	19-21	22-24	25-30	31-33	34-36
Assessment point (T)	-28 to - 10	-9 to -7	-6 to -4	-3 to -1	T0	1 to 3	4 to 6	7 to 9	10 to 12	13 to 15		
Study 1	Pre-intervention data collection				CO-OP KT	Post-intervention data collection					Analysis, reporting	
Study 2				Baseline		Post			Follow-up			
Study 3		Recruit patients: Historical control				Recruit patients: Intervention			Finish follow-up assessments			

Figure 3: Project Design and Timeline

References

1. Sharp S, Linkewich E, Willems J, Tahair N, Levy C, Bayley M. What should really be happening to our stroke patients post-acute care?: A system best practice model for inpatient rehab. Stroke 2014;45(Suppl 1).

2. Cicerone KD, Langenbahn DM, Braden C, Malec JF, Kalmar K, Fraas M, Felicetti T, Laatsch L, Harley JP, Bergquist T, et al. Evidence-based cognitive rehabilitation: Updated review of the literature from 2003 through 2008. Arch Phys Med Rehabil 2011 Apr;92(4):519-30.

3. Melkas S, Jokinen H, Hietanen M, Erkinjuntti T. Poststroke cognitive impairment and dementia: Prevalence, diagnosis, and treatment. Degenerative Neurological & Neuromuscular Disease 2014;4:21-27.

4. Cognitive rehab: A grounding in what is and what could be document Abstract presented at VasCog, Congress of the International Society for Vascular Behavioural and Cognitive Disorders. June 25, 2013]. .

5. Polatajko HJ, Mandich A. Enabiling occupation in children: The cognitive orientation to daily occupational performance (CO-OP) approach. First edition ed. Ottawa, Canada: CAOT Publications ACE; 2004. .

6. Eskes, G. on behalf of the Canadian Stroke Best Practices and Standards Working Group. Mood and cognition in patients following stroke. In: M. P. Lindsay, G. Gubitz, M. Bayley, S. Phillips, editors. Canadian stroke best practice recommendations 4th Edition ed. Ottawa, Ontario Canada: Canadian Stroke Network; 2013. .

7. Toronto Stroke Networks [Internet]: Toronto Stroke Networks; c2013 [cited 2014 10/07]. Available from: http://www.tostroke.com/.

8. Linkewich E, Khan F, Hall R. Using scarce stroke care resources for the greatest impact: Examining ontario's stroke report card Stroke 2014;45(Suppl 1).

9. sharp S, Willems J, Linkewich E, Tahiar N, Levy C, Bayley M. Facilitating best practices in rehabilitation for persons with stroke: Use of a triage tool in toronto. Stroke 2014;45(Suppl 1).

10. Fortin J, Skrabka K, Avinoam G, Willems J, Sharp S, Linkewich E. Developing excellence in stroke care through knowledge building and interprofessional collaborative processes. Stroke 2014;45(Suppl 1).

11. Fortin J, Skryabka K, Avinoam G, Sharp S, Willems J, Linkewich E. Developmental evaluation enhances utility of the toronto stroke networks virtual community of practice. Stroke 2014;45(Suppl 1).

12. Linkewich E. The toronto stroke networks virtual community of practice: Collaborative change leadership to create enhanced purpose for best practice implementation. Stroke 2013;44(ATP364).

13. Linkewich E, Willems J, Sharp S, Levy C, Bayley M. Importance of collaboration of hospital leaders in implementing stroke best practice across 17 organizations. Stroke 2013;44(ATP364).

14. Graham ID, Logan J, Harrison MB, Straus SE, Tetroe J, Caswell W, Robinson N. Lost in knowledge translation: Time for a map?. J Contin Educ Health Prof 2006;26(1):13-24.

15. Canadian Interprofessional Health Collaborative. A national interprofessional competency framework. 2010.

16. Ankolekar S, Renton C, Sare G, Ellender S, Sprigg N, Wardlaw JM, Bath PM, ENOS Trial Investigators. Relationship between poststroke cognition, baseline factors, and functional outcome: Data from "efficacy of nitric oxide in stroke" trial. J Stroke Cerebrovasc Dis 2014 Aug;23(7):1821-9.

17. McEwen SE, Polatajko HJ, Huijbregts MP, Ryan JD. Exploring a cognitive-based treatment approach to improve motor-based skill performance in chronic stroke: Results of three single case experiments. Brain Inj 2009 Dec;23(13-14):1041-53.

18. McEwen SE, Polatajko HJ, Huijbregts MPJ, Ryan JD. Inter-task transfer of meaningful, functional skills following a cognitive-based treatment: Results of three multiple baseline design experiments in adults with chronic stroke. Neuropsychological Rehabilitation 2010;20(4):541-61.

19. McEwen S, Polatajko H, Baum C, Rios J, Cirone D, Doherty M, Wolf T. Combined cognitive-strategy and task-specific training improves transfer to untrained activities in sub-acute stroke: An exploratory randomized controlled trial. Neurorehabilitation and Neural Repair in press.

20. Skidmore ER, Holm MB, Whyte EM, Dew MA, Dawson D, Becker JT. The feasibility of meta-cognitive strategy training in acute inpatient stroke rehabilitation: Case report. Neuropsychol Rehabil 2011 Apr;21(2):208-23.

21. Skidmore ER, Dawson DR, Whyte EM, Butters MA, Dew MA, Grattan ES, Becker JT, Holm MB. Developing complex interventions: Lessons learned from a pilot study examining strategy training in acute stroke rehabilitation. Clin Rehabil 2014 Apr;28(4):378-87.

22. Davis D, Evans M, Jadad A, Perrier L, Rath D, Ryan D, Sibbald G, Straus S, Rappolt S, Wowk M, et al. The case for knowledge translation: Shortening the journey from evidence to effect. Bmj 2003 Jul 5;327(7405):33-5.

23. National Collaborating Centre for Methods and Tools. Knowledge translation initiatives using a systems approach. Hamilton, ON: McMaster University; 2013. .

24. Kitson AL. The need for systems change: Reflections on knowledge translation and organizational change. J Adv Nurs 2009 Jan;65(1):217-28.

25. Health Quality Ontario & Ministry of Health and Long-Term Care. Quality-based procedures clinical handbook for stroke. Ontario, Canada: Queen's Printer for Ontario; 2013.

26. Dawson DR, Gaya A, Hunt A, Levine B, Lemsky C, Polatajko HJ. Using the cognitive orientation to occupational performance (CO-OP) with adults with executive dysfunction following traumatic brain injury. Can J Occup Ther 2009 Apr;76(2):115-27.

27. Henshaw E, Polatajko H, McEwen S, Ryan JD, Baum CM. Cognitive approach to improving participation after stroke: Two case studies. Am J Occup Ther 2011 Jan-Feb;65(1):55-63.

28. Polatajko HJ, McEwen SE, Ryan JD, Baum CM. Pilot randomized controlled trial investigating cognitive strategy use to improve goal performance after stroke. Am J Occup Ther 2012 Jan-Feb;66(1):104-9.

29. Penfold RB, Zhang F. Use of interrupted time series analysis in evaluating health care quality improvements. Acad Pediatr 2013 Nov-Dec;13(6 Suppl):S38-44.

30. Guide for the uniform data set for medical rehabilitation (including the FIM instrument). Version 5.1 ed. Buffalo, NY: State University of New York at Buffalo; 1997. .

31. Foy R, Eccles MP. Audit and feedback interventions. In: S. E. Straus, J. Tetroe, I. D. Graham, editors. Knowledge translation in health care: Moving from evidence to practice. Chichester, UK: John Wiley & Sons, Ltd; 2013. .

32. Korner-Bitensky N, Wood-Dauphinee S, Siemiatycki J, Shapiro S, Becker R. Health-related information postdischarge: Telephone versus face-to-face interviewing. Arch Phys Med Rehabil 1994 Dec;75(12):1287-96.

33. Segal ME, Schall RR. Determining functional/health status and its relation to disability in stroke survivors. Stroke 1994 Dec;25(12):2391-7.

34. Nasreddine ZS, Phillips NA, Bedirian V, Charbonneau S, Whitehead V, Collin I, Cummings JL, Chertkow H. The montreal cognitive assessment, MoCA: A brief screening tool for mild cognitive impairment. J Am Geriatr Soc 2005 Apr;53(4):695-9.

35. Computer Program to Calculate Sample Size Requirement to Compare 2 Proportions [Internet]: STATSTODO TRADING PTY LTD; c2014 [cited 2014 10/07]. Available from: https://www.statstodo.com/SSiz2Props_Pgm.php.

36. Borckardt JJ, Nash MR, Murphy MD, Moore M, O'Neil P. Clinical practice as natural laboratory for psychotherapy research. American Psychologist 2008;63(2):77-95.

37. Hsieh HF, Shannon SE. Three approaches to qualitative content analysis. Qual Health Res 2005 Nov;15(9):1277-88.

38. Law M, Polatajko H, Pollock N, Mccoll MA, Carswell A, Baptiste S. Pilot testing of the canadian occupational perforamnce measures: Clinical and measurement issues. Canadian Journal of Occupational Therapy Oct 1994;61(4):191-7.

39. Blanton S, Morris DM, Prettyman MG, McCulloch K, Redmond S, Light KE, Wolf SL. Lessons learned in participant recruitment and retention: The EXCITE trial. Phys Ther 2006 Nov;86(11):1520-33.

40. McEwen SE, Polatajko HJ, Davis JA, Huijbregts MPJ, Ryan JD. "*There's a real plan here, and I am responsible for that plan"*: participant experiences with a novel cognitive-based treatment approach for adults living with chronic stroke. . Disability and Rehabilitation 2010;32(7):540-50.
